# The Story of the Insane from Year to Year

**Published:** 1900-01-06

**Authors:** 


### THE STORY OF THE INSANE FROM YEAR TO YEAR.

TWELFTH SERIES.

### FIFE AND KINROSS ASYLUM.

IN this asylum the increase of patients at the end of the year was only 3, there being a total of 519 in residence. There were 83 first admissions, 33 readmissions, 38 recoveries, 34 discharged relieved, and 41 deaths. Calculated on the admissions, the recoveries were 31·81 per cent.; and the deaths, calculated on the average number resident, were 5·31 per cent. Cerebral and spinal diseases account for 21 of the deaths, pneumonia for 1, consumption for 7, and typhoid fever for 1. Ten of the admissions of the year owed their insanity to moral causes, 16 to intemperance in drink, 47 to hereditary influence, and 36 to previous attacks. Apparently, the typhoid fever was limited to the fatal case, and Dr. Turnbull does not give us any idea of the way the disease was introduced into the asylum. There are at present 25 vacant beds, and it is much to be desired that the committee will utilise the breathing space thus afforded to provide additions to the building, otherwise there is trouble ahead for them. The weekly cost of maintenance is 8s. 5½d. per week.

### THE DEVON COUNTY ASYLUM.

ON January 1st, 1898, this asylum contained 1,100 patients, and on December 31st the number was 1,108. There were 117 first admissions, 29 readmissions, 44 recoveries, 30 discharged relieved, and 57 deaths. Calculated in the usual way, the percentage of recoveries was 30·82, and that of the deaths was 5·10. The recoveries are rather low and the deaths very low. Of the year's deaths 25 were caused by cerebral and spinal diseases, 6 by consumption, 2 by pneumonia, and 1 by enteritis. Of the admissions, 26 owed their insanity to moral causes, 18 to intemperance in drink, and 44 to hereditary tendency. The asylum is practically full, and the difficulty of providing room for fresh cases was great, but extensions are to be built at a cost of £11,000. The medical superintendent, Dr. Saunders, resigned during the year, and was granted a pension of £742 10s. a year. "He bids to one and all a hearty farewell, accompanied by sincerest wishes for the continued prosperity of the institution," and the committee "express their sense of the loss which the patients suffer by the resignation of one whose assiduity for their welfare has been constant and unfailing." We hope that Dr. Saunders will long live to enjoy his well-merited pension. The medical staff now consists of a medical superintendent (Dr. Davis) and two assistant medical officers, but the Commissioners in Lunacy do not think this sufficient, and recommend the staff to be increased. No doubt the majority of the patients are healthy demented cases; the death-rate has been low for many years, which shows that the amount of sickness is not great, and it is difficult to see what a third assistant could find to do without spoiling one of the others. The weekly rate of maintenance was 8s. 8⅝d.

### MIDDLESBROUGH BOROUGH ASYLUM.

THIS is the first annual report, and it contains, in addition to the usual reports and statistics, a carefully drawn out description of the asylum and many illustrations. There were 182 first admissions, 58 readmissions, 8 recoveries, and 12 deaths. On the last day of 1898 215 patients remained in the asylum. Various moral causes account for 20 of the year's admissions, intemperance in drink accounts for 36, hereditary influence for 11, and previous attacks for 19. Of the deaths 7 were caused by cerebral disease, 1 by consumption, and 1 by enteritis. The first year of an asylum is always a time of great anxiety to the medical superintendent, and Dr. Pope is to be congratulated on the smoothness with which the machine has worked. There were no casualties of any kind, and it was not considered necessary to employ either restraint or seclusion. The total accommodation is for 250 patients, and Table I. states that 215 were in residence at the end of the year. Probably the chairman's report was written some little while afterwards, for he gives the number at 223, and adds that there are only 54 vacant beds, and this estimate includes the out-county cases. It seems clear that the asylum is not large enough for the district, and that a second mass of bricks and mortar will be in existence soon after the first has been cleared away. The Chairman said that all articles of consumption are purchased by contract from tradesmen in the town. In our opinion they should be purchased wherever they are cheapest and best. Any other system possesses a great element of danger, namely, impoverishing the whole of the ratepayers to enrich three or four of them. We trust the committee will alter their system. They say that Dr. Pope has "discharged the tedious and onerous duties of furnishing and equipping the asylum in a truly economical manner." The rate of maintenance is 14s. per head per week. This is high, but new asylums, especially when they are small, are sure to be above the average.

### STAFFORD COUNTY ASYLUM AT LICHFIELD.

ON the last day of December, 1898, there were in this asylum 829 patients, being an increase of 47 during the year. There were 265 first admissions, 34 readmissions, 96 recoveries, and 124 deaths. Calculated on the admissions the recoveries stand at 38·55 per cent., and the deaths at 15·25 per cent. on the average number resident. The former percentage is about the average in county asylums, but the latter is much above it, and Dr. Spence does not give any explanation of this high mortality which has lasted for several years. We notice that 60 of the deaths were caused by cerebral and spinal diseases, 27 by consumption, and 2 by pneumonia. Of the year's admissions 40 were driven insane by moral causes, 35 by drink, and 29 by hereditary influence. Considerable additions are being made to the asylum, and workmen have been in evidence during the whole of the year. The report contains nothing of a special nature. The committee "desire to record their confidence in the able administration of the asylum by Dr. Spence." The weekly cost of maintenance is 8s. 5⅜d. per head.

